# *Salmonella* virulence factors induce amino acid malabsorption in the ileum to promote ecosystem invasion of the large intestine

**DOI:** 10.1073/pnas.2417232121

**Published:** 2024-11-15

**Authors:** Lauren C. Radlinski, Andrew W. L. Rogers, Lalita Bechtold, Hugo L. P. Masson, Henry Nguyen, Anaïs B. Larabi, Connor R. Tiffany, Thaynara Parente de Carvalho, Renée M. Tsolis, Andreas J. Bäumler

**Affiliations:** ^a^Department of Medical Microbiology and Immunology, School of Medicine, University of California at Davis, Davis, CA 95616

**Keywords:** *Salmonella*, colonization resistance, short-chain fatty acids, microbiota

## Abstract

The gut microbiota produces high concentrations of antimicrobial short-chain fatty acids (SCFAs) that restrict the growth of invading microorganisms. The enteric pathogen *Salmonella enterica* serovar (*S.*) Typhimurium triggers inflammation in the large intestine to ultimately reduce microbiota density and bloom, but it is unclear how the pathogen gains a foothold in the homeostatic gut when SCFA-producing commensals are abundant. Here, we show that *S.* Typhimurium invasion of the ileal mucosa triggers malabsorption of dietary amino acids to produce downstream changes in nutrient availability in the large intestine. In gnotobiotic mice engrafted with a community of 17 human *Clostridia* isolates, *S.* Typhimurium virulence factors triggered marked changes in the cecal metabolome, including an elevated abundance of amino acids. In an ex vivo fecal culture model, we found that two of these amino acids, lysine and ornithine, countered SCFA-mediated growth inhibition by restoring *S.* Typhimurium pH homeostasis through the inducible amino acid decarboxylases CadA and SpeF, respectively. In a mouse model of gastrointestinal infection, *S.* Typhimurium CadA activity depleted dietary lysine to promote cecal ecosystem invasion in the presence of an intact microbiota. From these findings, we conclude that virulence factor–induced malabsorption of dietary amino acids in the small intestine changes the nutritional environment of the large intestine to provide *S.* Typhimurium with resources needed to counter growth inhibition by microbiota-derived SCFAs.

## Significance

The microbiota protects the host from microorganisms that cause disease in unprotected or immunocompromised individuals. Enteric pathogens such as *Salmonella enterica* serovar (*S.*) Typhimurium are adept at circumventing and weakening these protections and in doing so render the host susceptible to infection. Here, we identify a strategy by which *S*. Typhimurium uses its virulence factors to manipulate the host environment in the small intestine to trigger downstream changes in the environment of the large intestine that enable the pathogen to overcome microbiota-mediated defenses. The more general implications of our work are that ileitis-induced malabsorption causes downstream changes in microbial growth conditions in the large intestine, which can trigger compositional changes.

The gut microbiota is a critical frontline barrier that precludes the expansion of invading microorganisms through the production of antimicrobial compounds and the depletion of essential nutrients (1). During homeostasis, obligately anaerobic bacteria dominate the microbiota of the large intestine and ferment unabsorbed carbohydrates to produce high luminal concentrations of the short-chain fatty acids (SCFAs) acetate, propionate, and butyrate. These SCFAs are weak acids that become protonated in mildly acidic environments (HAc), such as the lumen of the colon (pH 5.7 to 6.2) (2), as the pH approaches the respective negative base-10 logarithm of the acid dissociation constant (pKa) for each molecule (~pH 4.7). Protonated SCFA are membrane permeable, but exposure to a more neutral pH in the cytosol (pH 7.2 to 7.8) (3–5) results in their dissociation into the salt and a proton (Ac^−^ + H^+^). The consequent acidification of the bacterial cytosol results in growth inhibition and serves as a canonical, nonspecific defense mechanism against invading enteric pathogens such as *Salmonella enterica* serovar Typhimurium (*S.* Typhimurium) (3, 6–8).

*S.* Typhimurium uses its virulence factors, two type III secretion systems (T3SS-1 and T3SS-2) (9, 10) encoded by *Salmonella* pathogenicity island (SPI)1 and SPI2 (11, 12), respectively, to break colonization resistance through mechanisms that are not fully resolved (13, 14). T3SS-1 and T3SS-2 trigger intestinal inflammation (15–17), which boosts growth of *S.* Typhimurium by increasing the availability of host-derived respiratory electron acceptors, including tetrathionate (18), nitrate (19, 20), and oxygen (21). In addition, aspartate is liberated when phagocyte-derived reactive oxygen species lyse luminal bacteria (22), which fuels growth of *S.* Typhimurium through fumarate respiration (23). Tetrathionate respiration has been shown to promote growth of *S.* Typhimurium in the lumen of the murine cecum by utilizing ethanolamine (24), which is generated when taurine liberated during deconjugation of bile acids is used as an electron acceptor by *Deltaproteobacteria*. Oxygen and nitrate enable the pathogen to utilize host-derived lactate (25) or 1,2-propanediol (26), a microbiota-derived fermentation product of pentoses. However, growth during in vitro culture under conditions that mimic the cecal environment suggests that high concentrations of SCFAs and the acidic environment of the cecum counter the competitive edge that oxygen and nitrate respiration confer upon the pathogen (27). These data suggest that *S.* Typhimurium virulence factors act on the host to generate yet unidentified resources that enable the pathogen to overcome growth inhibition by SCFAs in the lumen of the large intestine.

Here, we used untargeted metabolomics to identify resources generated by *S.* Typhimurium virulence factor activity during gastrointestinal infection and investigated their role in countering SCFA-mediated intracellular acidification.

## Results

### *Salmonella* Virulence Factors Increase the Cecal Concentrations of Several Amino Acids.

**Fig. 1.**
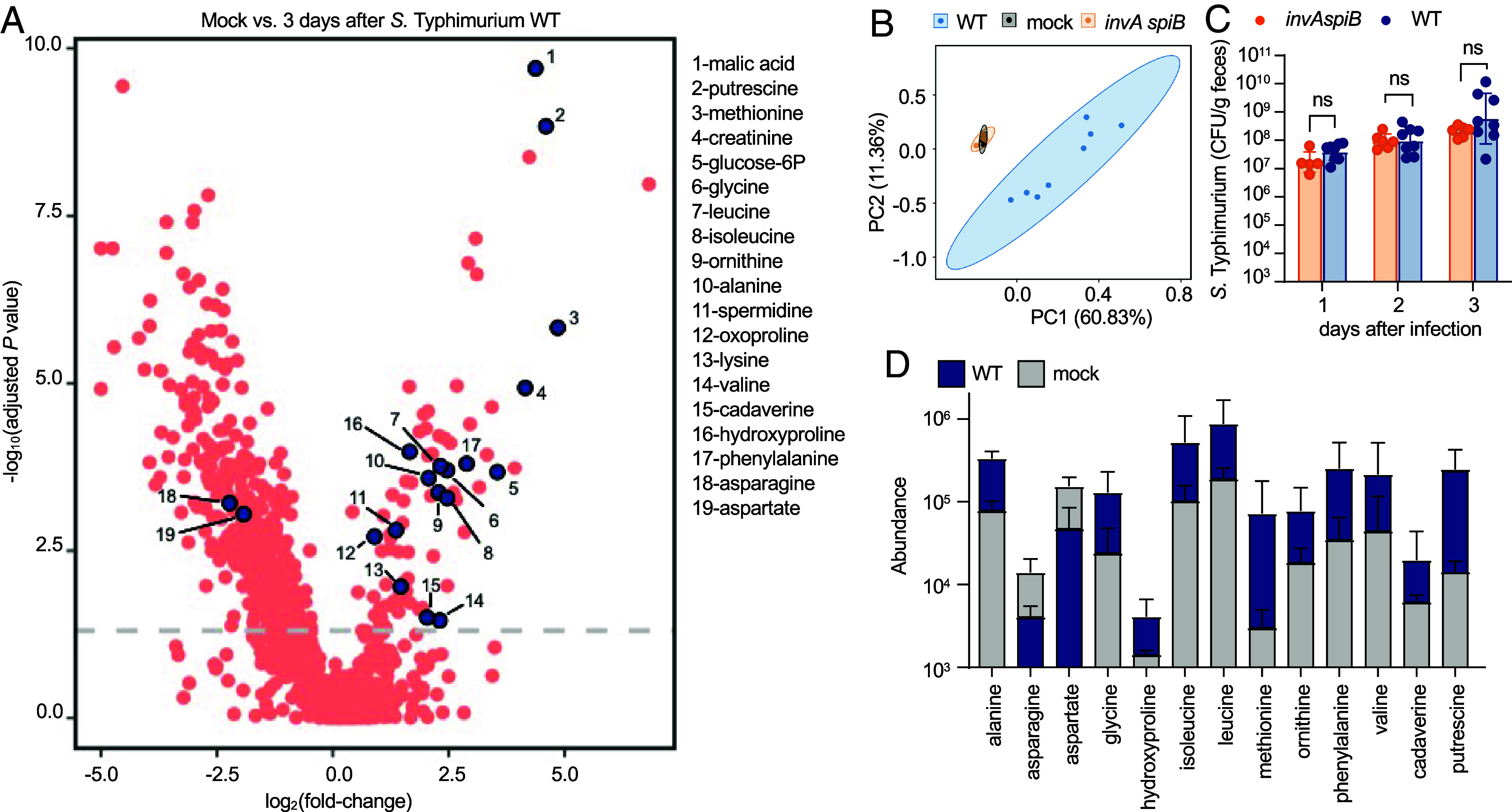
*S.* Typhimurium virulence factors change the cecal metabolome. Germ-free Swiss Webster mice were engrafted with a defined microbial consortium consisting of 17 human *Clostridia* isolates(29). One week later, mice were mock infected (mock) (*N* = 5), infected with the *S.* Typhimurium WT (*N* = 8), or with an avirulent *S.* Typhimurium *invA spiB* mutant (*invA spiB*) (*N* = 6). Cecal contents were collected 3 d after infection for untargeted metabolomics analysis. (*A*) Volcano blot showing metabolite abundance in the ceca of mock vs WT-infected mice. The *Y* axis shows the decadic logarithm of the false discovery rate (FDR)-corrected *P* value. The dashed line is set at an FDR-corrected *P* value of 0.05. Metabolites with a negative fold-change value decreased in mice infected with *S.* Typhimurium, while metabolites with a positive fold-change value increased. (*B*) Principal component analysis of the cecal metabolome in the indicated groups of mice. Ovals indicate the 95% CI. (*C*) The intestinal burden of *S.* Typhimurium was tracked for 3 d by enumerating colony-forming units (CFU) of *S.* Typhimurium in the feces (days 1 and 2) or cecal content (day 3). (*B* and *C*) Each dot represents data from one animal. (*D*) The graphs show the abundance of the indicated metabolites in mock-infected mice compared to mice infected with the *S.* Typhimurium WT. (*C* and *D*) Bars represent geometric means ± SE. ns, not significant (Mann–Whitney).

Mutations in *invA* and *spiB* result in inactivation of T3SS-1 and T3SS-2, respectively, which can be used to study how virulence factors contribute to the interaction of *S.* Typhimurium with its host (18, 21). To investigate how virulence factors change the nutritional landscape of the gut, we compared cecal contents after mock infection or infection with either the virulent *S.* Typhimurium wild type (WT) or an avirulent *invA spiB* mutant. To avoid the possibility that variation in the microbiota composition would confound our results, gnotobiotic Swiss Webster mice that had been engrafted one week earlier with a defined microbial community consisting of 17 human *Clostridia* isolates (28, 29) were used for infection experiments. Three days after infection of gnotobiotic mice, the soluble fraction of the cecal content was collected, filter sterilized, and analyzed by untargeted gas chromatography time-of-flight mass spectrometry (metabolite profiling). The concentration of only five metabolites was significantly changed in gnotobiotic mice infected with the avirulent *invA spiB* mutant compared to gnotobiotic mock-infected mice (*SI Appendix*, Fig. S1*A*). In contrast, numerous changes in metabolite concentrations were observed during infection with the *S.* Typhimurium WT compared to mock infection (Fig. 1*A*). Principal component analysis suggested that metabolite profiles of mock-infected gnotobiotic mice and gnotobiotic mice infected with an avirulent *invA spiB* mutant were similar, whereas a distinct clustering was observed for cecal contents collected from gnotobiotic mice infected with the virulent *S.* Typhimurium WT (Fig. 1*B*). Since a simple consortium of 17 *Clostridia* isolates is not sufficient to confer colonization resistance (30), the *S.* Typhimurium WT or an *invA spiB* mutant were recovered in similar numbers from the feces (Fig. 1*C*). Thus, differences in cecal metabolite profiles triggered by infection with the virulent *S.* Typhimurium WT compared to infection with an avirulent *invA spiB* mutant (*SI Appendix*, Fig. S1*B*) were not due to differences in pathogen burden (Fig. 1*C*). These data suggested that *S.* Typhimurium virulence factors triggered marked changes in the composition of the murine cecal metabolome.

Among metabolites with significantly elevated abundance during infection with the *S.* Typhimurium WT compared to mock infection (Fig. 1*A*) or to infection with an *invA spiB* mutant (*SI Appendix*, Fig. S1*B*) were several amino acids. Specifically, infection with the *S.* Typhimurium WT increased the cecal concentrations of alanine, glycine, isoleucine, leucine, lysine, methionine, ornithine, phenylalanine, and valine (Fig. 1*D* and *SI Appendix*, Fig. S2). These data suggested that *S.* Typhimurium virulence factors increased the availability of several amino acids in the murine cecum through an unknown mechanism.

### Virulence Factors Trigger Malabsorption of Dietary Amino Acids in the Small Intestine.

Dietary amino acids are absorbed in the small intestine, which limits their availability in the large intestine. However, conditions of inflammation in the small intestine can induce malabsorption of amino acids (31, 32). *S.* Typhimurium initiates infection by using its virulence factors to invade Peyer’s patches in the ileum (33), which triggers inflammation in the small intestine (34). We thus hypothesized that *S.* Typhimurium–induced inflammation causes malabsorption of amino acids in the small intestine.

**Fig. 2.**
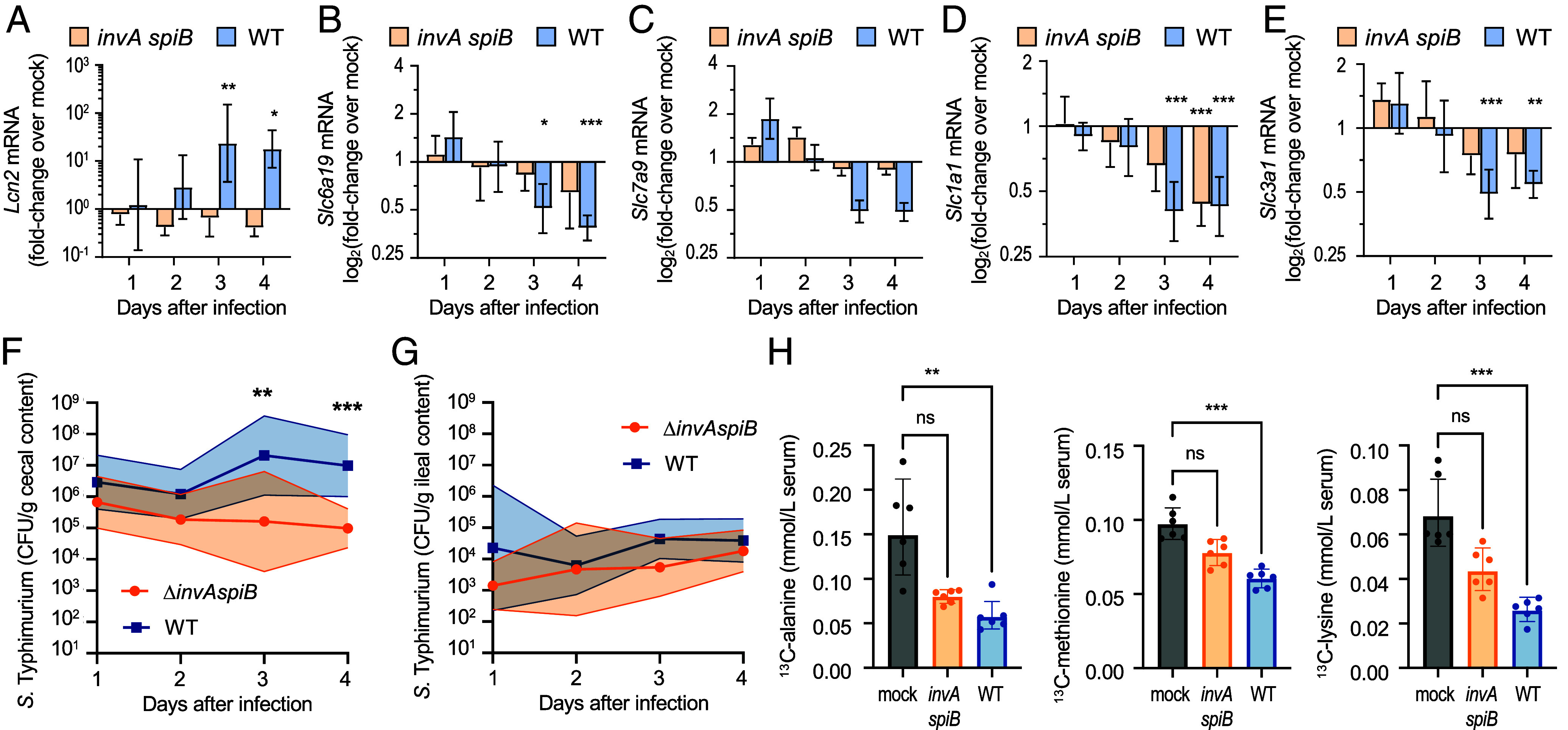
*S.* Typhimurium virulence factors trigger malabsorption of amino acids. (*A*–*G*) Groups of CBA/J mice (*N* = 8) were mock infected (mock) or infected with 10^9^ CFU of the *S.* Typhimurium WT or an isogenic *S.* Typhimurium *invAspiB* mutant (*invA spiB*). At the indicated timepoints, groups of 8 mice were killed to collect samples. (*A*–*E*) Ileal epithelial cells were isolated for extraction of host mRNA. Fold changes in transcript levels of *Lcn2* (*A*), *Slc6a19* (*B*), *Slc7a9* (*C*), *Slc1a1* (*D*), and *Slc3a1* (*E*), were determined by qRT-PCR. Graphs show geometric mean ± SE. (*A*) The 3-d time point for *Lcn2* mRNA elicited by infection with the *S.* Typhimurium WT has been published previously (42). (*F* and *G*) *S.* Typhimurium CFU were determined in cecal (*F*) or ileal (*G*) contents. Thick lines and symbols indicate the geometric mean. Thin lines indicate the SD. (*H*) Groups of CBA/J mice (*N* = 6) were mock infected or infected with WT or *invA spiB*. Four days later, mice were orally inoculated with a mixture of ^13^C-labeled alanine, ^13^C-labeled methionine, and ^13^C-labeled lysine, and serum samples were collected 30 min later. The graphs show the serum concentrations of the indicated ^13^C-labeled amino acids in the indicated groups. Bars represent geometric means ± SE. (*A*–*E* and *H*) Kruskal–Wallis; (*F*) Mann–Whitney; *P < 0.05; **P < 0.01; ***P < 0.001; ns, *P* > 0.05.

To test this idea, genetically resistant (CBA/J) mice with an intact microbiota were mock infected or infected with either the virulent *S.* Typhimurium WT or an avirulent *invA spiB* mutant. Groups of mice were killed daily for 4 d to establish a time course. Expression of *Lcn2*, encoding the proinflammatory marker lipocalin-2, was significantly elevated in mRNA isolated from preparations of ileal epithelial cells starting at 3 d after infection with the *S.* Typhimurium WT, but not after infection with the avirulent *invA spiB* mutant (Fig. 2*A*). Elevated *Lcn2* transcript levels correlated with a decreased expression of the *Slc6a19*, *Slc3a1*, *Slc7a9*, and *Slc1a1* genes, which encode amino acid transporters that are synthesized in ileal enterocytes (35) (Fig. 2 *B*–*E*). Slc6a19 and Slc1a1 transport glycine and glutamate, respectively, whereas Slc3a1 and Slc7a9 are both cationic and neutral amino acid transporters responsible for absorbing cystine and dibasic amino acids, such as lysine, ornithine, and arginine (35, 36). The onset of increased *Lcn2* expression in ileal epithelial cells coincided with increased recovery of the *S.* Typhimurium WT from cecal contents compared to the *invA spiB* mutant (Fig. 2*F*). In contrast, the WT and *invA spiB* mutant were recovered in similar numbers from ileal contents (Fig. 2*G*). Scoring of sections from the ileum collected 4 d after infection revealed inflammatory changes in mice infected with the *S.* Typhimurium WT, but not in mock-infected mice or mice infected with the *invA spiB* mutant (*SI Appendix*, Fig. S3).

To directly test whether *S.* Typhimurium infection triggers malabsorption of dietary amino acids in the small intestine, CBA/J mice were mock infected or infected with either the virulent *S.* Typhimurium WT or an avirulent *invA spiB* mutant. Four days later, mice were orally inoculated with a mixture of ^13^C-labeled alanine, ^13^C-labeled methionine, and ^13^C-labeled lysine. Absorption of ^13^C-labeled dietary amino acids was determined by measuring their concentration in serum 30 min later. The concentrations of ^13^C-labeled amino acids in serum were significantly reduced in mice infected with the virulent *S.* Typhimurium WT compared to mock-infected mice, suggesting that the pathogen induced malabsorption (Fig. 2*H*). The pathogen required virulence factors to trigger malabsorption of dietary amino acids because significantly reduced serum levels of ^13^C-labeled amino acids were not observed after infection with the *S.* Typhimurium *invA spiB* mutant. Although a significant reduction was not observed, the concentrations of ^13^C-labeled amino acids trended lower in serum of mice infected with the *S.* Typhimurium *invA spiB* mutant compared to mock-infected mice. A possible explanation is that flagella provide an *invA*-independent pathway for invading ileal Peyer’s patches (37), but this was not further investigated.

Collectively, these findings suggested that *S.* Typhimurium virulence factors trigger inflammation in the small intestine, which induces malabsorption of dietary amino acids.

### Lysine and Ornithine Decarboxylation Counters Growth Inhibition by SCFAs In Vitro.

We reasoned that amino acids might be beneficial for the pathogen because their decarboxylation is a proton-consuming reaction that can counter SCFA-mediated intracellular acidification in the mildly acidic colon. *S.* Typhimurium encodes inducible decarboxylases for lysine (CadA) and ornithine (SpeF), two of the amino acids that exhibited an elevated cecal concentration during infection with the *S.* Typhimurium WT (Fig. 1*D* and *SI Appendix*, Fig. S1*B*). The decarboxylation of lysine and ornithine consumes one proton and generates the corresponding polyamines, cadaverine and putrescine, respectively, which are then excreted by their cognate polyamine-amino acid antiporters (CadB and PotE, respectively). CadB and PotE link the excretion of cadaverine and putrescine to the import of lysine and ornithine, respectively, to perpetuate this buffering reaction. Notably, putrescine and cadaverine were among the known metabolites with significantly elevated concentration in cecal contents of gnotobiotic mice infected with the *S.* Typhimurium WT compared to mock-infected gnotobiotic mice (Fig. 1*D*), supporting the idea that the pathogen decarboxylates the corresponding amino acids in vivo.

**Fig. 3.**
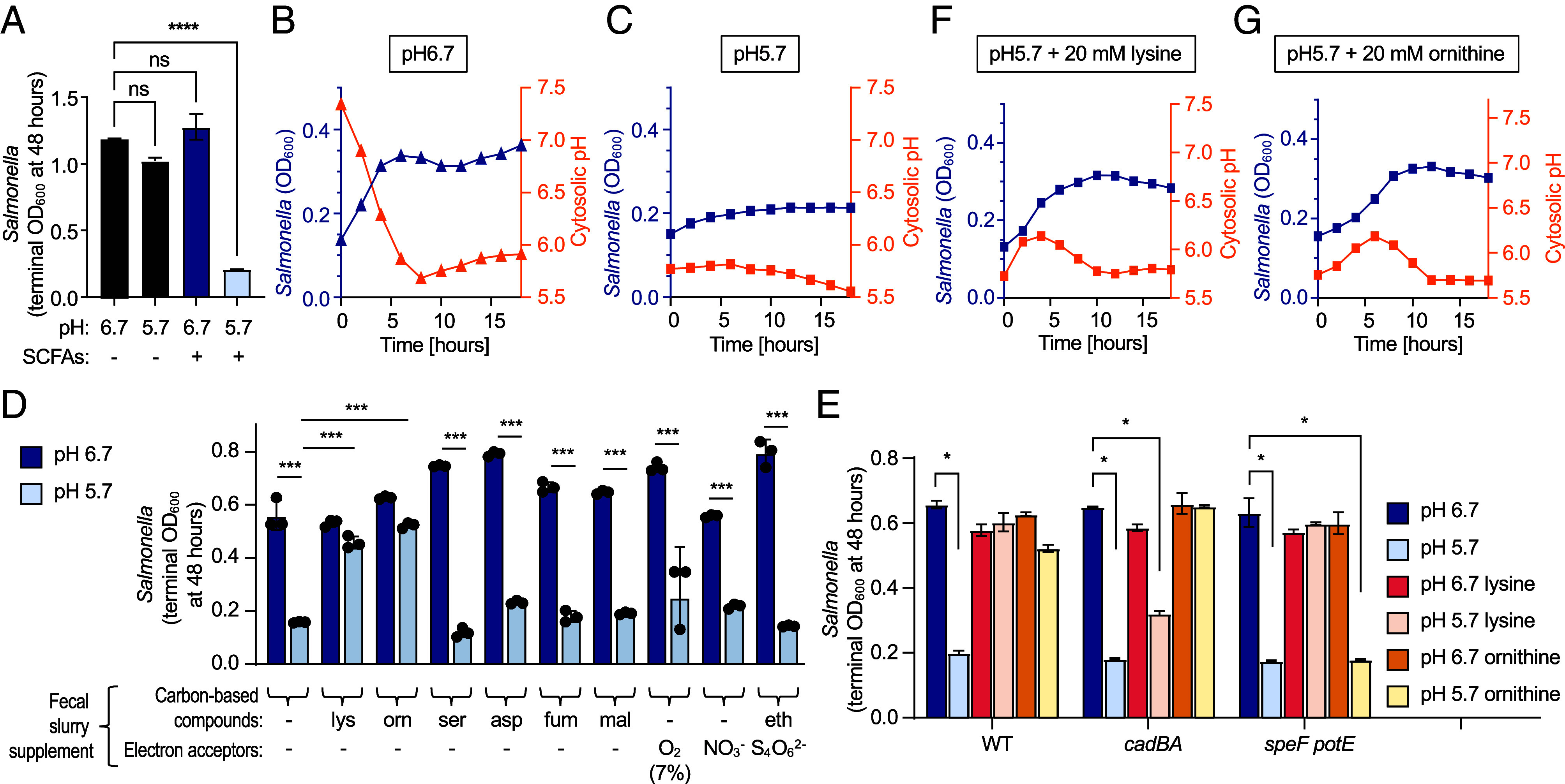
Decarboxylation of lysine and ornithine counters SCFA-mediated acidification of the bacterial cytosol. Filter-sterilized murine fecal homogenates were adjusted to the indicated pH and inoculated with the indicated *S.* Typhimurium strains. Each experiment was repeated three times. (A) Growth of *S.* Typhimurium at the indicated pH in the presence (+) or absence (–) of 50 mM acetate, 25 mM butyrate, and 6 mM propionate (SCFAs) was determined by measuring the optical density at 600 nm (OD_600_). Bars represent geometric mean ± SD. (*B*–*G*) *S.* Typhimurium was transformed with pGFPR01 to measure cytosolic pH and bacterial growth (OD_600_) in fecal homogenates supplemented with 50 mM acetate at pH 6.7 (*B*) or pH5.7 (*C*–*G*). *S.* Typhimurium growth and cytosolic pH were determined at pH 5.7 in the absence of amino acid supplementation (*C*) or after supplementation with lysine (*F*) or ornithine (*G*). (*D*) *S.* Typhimurium growth (OD_600_) 48 h after inoculation of SCFA-supplemented fecal homogenates at pH 6.7 or 5.7 in the presence of the indicated metabolites or respiratory electron acceptors. lys, 10 mM lysine; orn, 10 mM ornithine; ser, 10 mM serine; asp, 10 mM aspartate; fum, 10 mM fumarate; mal, 10 mM malate; eth, 10 mM ethanolamine; O_2_, 7% oxygen; NO_3_^−^, 20 mM nitrate; S_4_O_6_^2−^, 40 mM tetrathionate. (*E*) Growth (OD_600_) of the indicated *S.* Typhimurium strains in SCFA-supplemented fecal homogenates adjusted to pH 6.7 or 5.7 in the absence of amino acid supplementation or when supplemented with 20 mM lysine or 20 mM ornithine. *P < 0.05; ***P < 0.001; ****P < 0.0001; ns, not significant (one-way ANOVA with Dunnett’s multiple comparison test).

To test the hypothesis that amino acid decarboxylation helps *S.* Typhimurium to overcome growth inhibition by SCFAs, we modified a previously described fecal homogenate culture model (27). Murine fecal pellets were collected and homogenized in MES-buffered saline, centrifuged to remove insoluble materials, and titrated to pH 6.7 or 5.7. Filter-sterilized supernatants were equilibrated to oxygen concentrations that are representative of the large intestine (0.5 to 1%) in a hypoxia chamber. When prepared at 125 mg feces/mL, this medium supported robust *S.* Typhimurium growth that surpassed that obtained with rich laboratory medium (i.e., Luria-Bertani [LB] broth) (*SI Appendix*, Fig. S4). Endogenous SCFAs were insufficient to restrict *Salmonella* growth in medium acidified to pH 5.7 (Fig. 3*A*), presumably because their concentrations were lowered by dilution in saline and/or evaporation of these volatile compounds during processing. However, supplementation with 50 mM acetate, 25 mM butyrate, and 6 mM propionate, concentrations that are characteristic of the murine colon during homeostasis (27), resulted in growth inhibition of *S.* Typhimurium in fecal homogenate media adjusted to pH 5.7, but not to pH 6.7 (Fig. 3*A*).

Next, we monitored the pH in the bacterial cytosol using the pH sensor pHluorin (38). The two excitation peaks of pHluorin (λ405/λ488) are directly proportional to changes in pH, providing an opportunity to monitor changes in intracytoplasmic pH alongside growth over time. *S.* Typhimurium growth (OD_600_) and intracellular pH (pHluorin excitation at λ405/488) were measured simultaneously in fecal homogenate media in the presence of 50 mM acetate. In fecal homogenate media adjusted to pH 6.7, *S.* Typhimurium cytosolic pH acidified from approximately pH 7.3 to pH 5.9 during 16 h of growth (Fig. 3*B*). In contrast, in fecal homogenate media adjusted to pH 5.7 cytosolic pH instantly equilibrated to that of the culture media (pH 5.7), and growth was inhibited (Fig. 3*C*). These data from our ex vivo fecal homogenate model suggested that in an acidic environment (pH 5.7), SCFAs rapidly acidify the bacterial cytosol and inhibit growth of *S.* Typhimurium.

We then wanted to test whether the addition of lysine or ornithine would enable *S.* Typhimurium to overcome growth inhibition at pH 5.7 in fecal homogenate media supplemented with SCFAs (i.e., 50 mM acetate, 25 mM butyrate, and 6 mM propionate). For comparison, other resources previously implicated in growth of *S.* Typhimurium in the murine intestine were added to fecal homogenate media supplemented with SCFAs, which included L-aspartate (22, 23), fumarate (22, 23), malate (23), oxygen (21), nitrate (19, 20), or tetrathionate and ethanolamine (18, 24). Under these conditions, only L-lysine and L-ornithine rescued *S.* Typhimurium growth at pH 5.7 (Fig. 3*D*). Lysine-dependent rescue of growth at pH 5.7 was dependent on lysine decarboxylation because it was no longer observed with a *S.* Typhimurium *cadBA* mutant (Fig. 3*E*). Similarly, L-ornithine-dependent rescue of *S.* Typhimurium growth at pH 5.7 required ornithine utilization because it was no longer observed with a *speF potE* mutant. Collectively, these data suggested that decarboxylation of lysine and ornithine enabled *S.* Typhimurium to overcome growth inhibition by SCFAs.

To investigate whether lysine or ornithine could alleviate acidification of the bacterial cytosol by acetate at pH 5.7, we monitored intracellular pH using the pH sensor pHluorin (38). Addition of 20 mM L-lysine or L-ornithine increased *S.* Typhimurium cytosolic pH, which was accompanied by bacterial growth in the presence of 50 mM acetate at pH 5.7 (Fig. 3 *F* and *G*). After approximately 10 h of growth, cytosolic pH returned to ~5.7, presumably following the depletion of available L-lysine or L-ornithine, and this led to cessation of growth. Notably, cytosol alkalinization and the onset of exponential growth occurred more rapidly with the addition of 20 mM L-lysine compared to addition of 20 mM L-ornithine, suggesting that the pathogen might prefer lysine under these conditions.

### Decarboxylation of Dietary Lysine Facilitates Ecosystem Invasion by *Salmonella*.

**Fig. 4.**
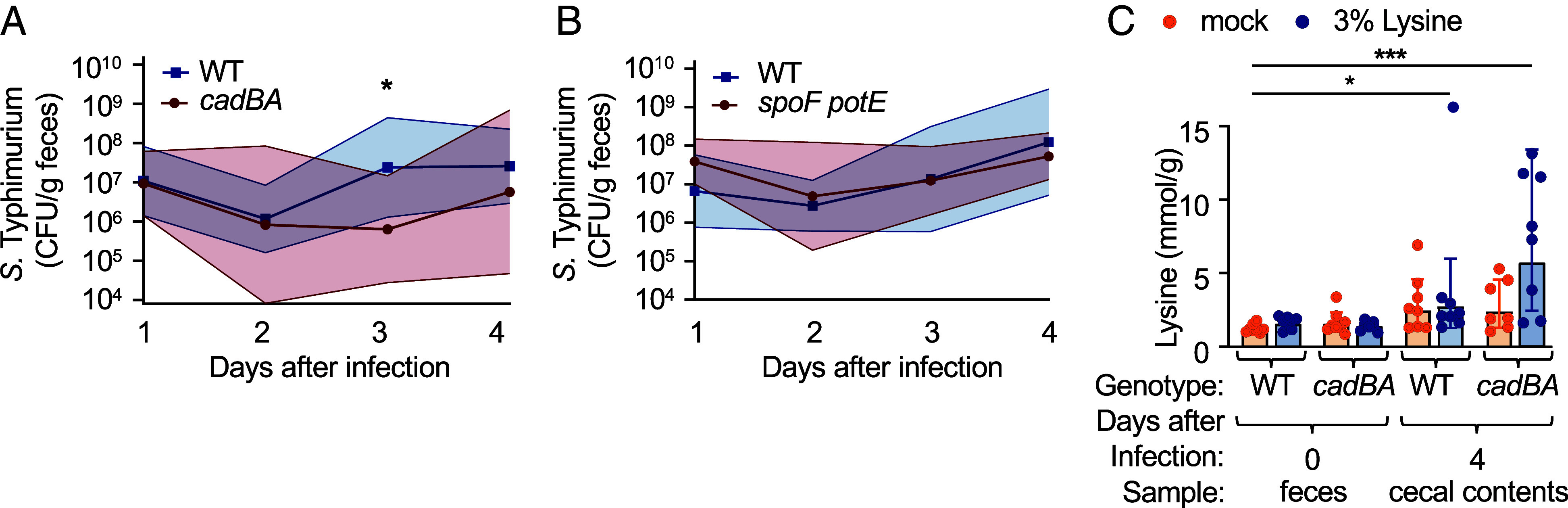
Malabsorption of dietary lysine promotes *cadBA*-mediated ecosystem invasion. (*A* and *B*) Groups of antibiotic-naïve CBA/J mice (*N* = 8) were infected with 10^9^ CFU of the *S.* Typhimurium WT or the indicated isogenic mutant strains by oral gavage. Thick lines and symbols indicate the geometric mean. Thin lines indicate the SD. (*C*) Groups of antibiotic-naïve CBA/J mice (*N* = 8) received regular drinking water (mock) or drinking water supplemented with 3% lysine. One day after the beginning of lysine supplementation, mice were infected with 10^9^ CFU of the *S.* Typhimurium WT or an isogenic *cadBA* mutant. The concentration of lysine was determined at the indicated time points after infection. Bars represent geometric mean ± SD. Each dot represents data from one animal. (A) Mann–Whitney; (H) Kruskal–Wallis; *P < 0.05; ***P < 0.001.

To determine whether decarboxylation of lysine and ornithine is important for colonizing the gastrointestinal tract in the presence of complex SCFA-producing bacterial community, genetically resistant (CBA/J) mice with an intact microbiota were infected with the *S.* Typhimurium WT, a *cadBA* mutant, or a *speF potE* mutant. Lysine decarboxylation was required for intestinal colonization as indicated by reduced recovery of the *cadBA* mutant compared to the *S.* Typhimurium WT from the feces at 3 d after infection (Fig. 4*A*). In contrast, ornithine decarboxylation did not appear to contribute appreciably to early *S.* Typhimurium colonization, as the fecal burden of a *speFpotE* mutant was similar to that of the isogenic WT (Fig. 4*B*).

The hypothesis that *S.* Typhimurium causes malabsorption in the ileum predicts that infection increases the concentration of dietary lysine in the feces of mice with a complex microbiota. To test this idea, genetically resistant (CBA/J) mice with an intact microbiota received regular drinking water or drinking water supplemented with 3% lysine and were infected 1 d later with the *S.* Typhimurium WT. Prior to infection, dietary supplementation with lysine did not increase the concentration of the amino acid in the feces, presumably because the dietary supplement was either fully absorbed in the small intestine and/or consumed by gut microbes (Fig. 4*C*). Four days after infection with the *S.* Typhimurium WT, dietary supplementation caused a small but significant increase the concentration of lysine in the feces. We considered two possible explanations for the small magnitude by which the concentration of lysine increased in the feces. One possibility was that *S.* Typhimurium infection does not markedly increase the concentration of dietary lysine in the large intestine. Alternatively, *S.* Typhimurium infection causes a marked increase in the concentration of dietary lysine in the large intestine, but this increase is masked by pathogen-mediated lysine decarboxylation. To distinguish between these two possibilities, the experiment was repeated with a mutant unable to decarboxylate lysine (*cadBA* mutant). Notably, dietary supplementation with lysine caused a robust and significant increase in the concentration of lysine in the feces of mice infected with a *cadBA* mutant. These data were consistent with the idea that an increased availability of dietary lysine in the large intestine is masked by *S.* Typhimurium-mediated decarboxylation of the amino acid.

Collectively, our data provide direct support for the idea that virulence factor–induced malabsorption in the small intestine (Fig. 2) increases in the concentration of dietary lysine in the cecal lumen (Figs. 1 *A* and *D* and 4*C*). The pathogen decarboxylates lysine using the *cadBA* genes to prevent acidification of the cytosol (Fig. 3*F*), which promotes its growth (Fig. 4*A*) at low pH in the presence of high concentrations of SCFAs (Fig. 3 *D* and *E*).

## Discussion

*S.* Typhimurium enters intestinal tissue predominantly in the ileum at areas of Peyer’s patches, whereas the bulk of the luminal pathogen population localizes to the large intestine (33). *S.* Typhimurium is thought to gain prominence in the lumen of the large intestine because a fraction of the pathogen population uses T3SS-1 and T3SS-2 to invade the cecal mucosa, which triggers acute mucosal inflammation (16, 17). Inflammation-induced changes in the environment of the large intestine then fuel growth of the remaining *S.* Typhimurium population in the cecal lumen (13), which promotes transmission of the pathogen by the fecal–oral route to the next susceptible host (14, 21). In calves, where the pathogen causes a self-limited gastroenteritis that closely mimics human infection (39, 40), the *S.* Typhimurium population that enters the intestinal mucosa is eventually cleared by the host’s inflammatory response (15). Thus, invasion of the intestinal mucosa appears to be a dead end for the pathogen, but in the cecal mucosa, this sacrifice is offset by the growth benefit mucosal inflammation confers upon the luminal *S.* Typhimurium population (13, 14). One problem with this model is that it does not explain why the pathogen preferentially invades the ileal mucosa (33). Here, we show that T3SS-1 and T3SS-2-mediated invasion and survival in the small intestinal mucosa triggers malabsorption of dietary lysine, which increases the concentration of this amino acid in the large intestine. A *cadBA*-dependent decarboxylation of lysine enables the pathogen to counter intracellular acidification by high concentrations of SCFAs that are present in the acidic environment of the cecal lumen. Collectively these results suggest that the apparent preference of *S.* Typhimurium for invading the ileal mucosa (33) is a mechanism that facilitates a downstream ecosystem invasion in the cecum, where high concentrations of SCFAs and a low pH restrict growth of new arrivals.

Previous studies show that cecal inflammation drives a dominance of *S.* Typhimurium in the cecal microbiota by generating electron acceptors to support fumarate respiration (22, 23), tetrathionate respiration (18), nitrate respiration (19, 20), and aerobic respiration (21, 25) of the pathogen. However, neither oxygen nor nitrate can counter growth inhibition by SCFAs under in vitro growth conditions that mimic the lumen of the cecum (27). Similarly, our data suggest that high concentrations of SCFAs and the acidic environment in the cecum counter the competitive advantage conferred upon *S.* Typhimurium through tetrathionate respiration or fumarate respiration in vitro. These data suggest that respiration is necessary but not sufficient to explain ecosystem invasion by the pathogen. Notably, at 3 d after infection, the time point at which the pathogen population starts to expand in the cecum, *S.* Typhimurium–induced inflammation does neither reduce the microbiota density (41) nor its composition (42). These data suggest that lysine decarboxylation is necessary for the initial invasion of an intact ecosystem, in which an undisturbed microbiota produces high concentrations of SCFAs. As the infection progresses (i.e., between days 7 and 10 after infection), *S.* Typhimurium–induced colitis decreases the microbiota density by two orders of magnitude (41), which is accompanied by a depletion of *Clostridia* species (43) and a marked reduction in SCFA concentrations (21). Under these conditions, respiratory electron acceptors are sufficient to fuel growth of the pathogen, and resources that counter cytosolic acidification by SCFAs might no longer be needed. A similar reduction in the microbiota density and reduction in SCFA concentrations is also observed after treatment with streptomycin (6, 21), which is often used to increase the susceptibility of mice to *S.* Typhimurium infection (44).

The picture emerging from these studies is that *S.* Typhimurium initiates infection by triggering malabsorption in the ileum, which facilitates invasion of the cecal ecosystem where high concentrations of SCFAs produced by an intact microbiota limit the initial growth of the pathogen. As the infection progresses, depletion of the cecal microbiota by host inflammatory responses creates more favorable growth conditions for the pathogen, resulting in intestinal domination by *S.* Typhimurium, which in turn ensures transmission by the fecal–oral route (14, 21).

## Materials and Methods

### Ethics Statement.

All mice used in the study were maintained under specific pathogen-free conditions in the Animal Association of Laboratory Animal Care-accredited University of California, Davis, Teaching and Research Animal Services. All protocols were approved by the Institutional Animal Care and Use Committee at the University of California, Davis, Protocol No. 22987, and all experiments were performed in accordance with the NIH. Animals were anesthetized by CO_2_ asphyxiation followed by cervical dislocation.

### Bacterial Strains and Growth Conditions.

*Escherichia coli* and *S*. Typhimurium strains used in this study are listed in *SI Appendix*, Table S1. *E. coli* and *S.* Typhimurium strains were cultured in Luria-Bertani (LB) broth (LB, BD Biosciences) or on LB plates unless otherwise indicated. Broth culture and plate incubation was carried out under atmospheric conditions at 37 °C for 16 to 20 h. C17 *Clostridial* isolates were grown anaerobically in Eggerth-Gagnon broth for at least 72 h. Agar plates and liquid media were supplemented with antibiotics used at the following concentrations when required: carbenicillin (Carb), 100 μg/mL; chloramphenicol (Cm), 30 μg/mL; and kanamycin (Kan), 100 μg/mL.

### Strain Construction.

*S.* Typhimurium IR715 deletion mutants were constructed by singly deleting the coding sequences of *cadBA* and *speFpotE* using primers listed in *SI Appendix*, Table S2. Briefly, flanking primers were designed to anneal approximately 500 base pairs upstream and downstream of the coding region. The resulting PCR product was inserted into plasmid pRE112 in accordance with the NEBuilder HiFi DNA Assembly protocol (New England Biolabs). Mutant alleles were integrated onto the chromosome of IR715 as described previously (45). Briefly, pRE112 containing the in-frame deletion and gene-specific flanking regions was mated into *S.* Typhimurium via *E. coli* S17-λpir. Primary integrants were selected with chloramphenicol and kanamycin, then grown for 2 h in LB without selection to allow for recombination. Dilutions of *S.* Typhimurium were plated on LB with 8% sucrose for counterselection (loss of plasmid). Deletion strains were confirmed through PCR and sequencing (Genewiz).

### Ex Vivo Fecal Homogenate Growth Assay.

Freshly voided fecal pellets were collected from CBA/J mice and stored at −80 °C. Fecal pellets were homogenized in 50 mM 2-(N-morpholino)ethanesulfonic acid (MES), 0.9 % NaCl at a concentration of 125 mg/mL with or without 50 mM sodium acetate, 6 mM sodium propionate, and 25 mM sodium butyrate. The resulting fecal homogenate was centrifuged at 10,000×*g* for 5 min to pellet particulate matter, then diluted to the stated concentration. When applicable, the fecal homogenate supernatant was supplemented with the stated concentration of metabolites. Prepared homogenates were corrected to a pH of 5.7 or 6.7 using HCl and NaOH and sterilized by filtration through a 0.2 μm filters.

Growth curves of *S.* Typhimurium in fecal homogenates were performed in 96-well plates containing 200 μL of fecal homogenate per well. Uninoculated plates were equilibrated to 0.7 to 1% O_2_ and 5% CO_2_ in a hypoxia chamber (Coy Laboratories) overnight. Overnight cultures of *S.* Typhimurium washed with sterile PBS were used to inoculate wells of fecal homogenate at a 1:100 dilution. The optical density at 600 nm of each well was measured every 30 min in a Victor Nivo multimode microplate reader (Perkin Elmer) at 37 °C and 0.5 to 1% O_2_ tension.

### Cytosolic pH Measurements Using pHluorin.

For measuring cytosolic pH, *S.* Typhimurium was transformed with the plasmid pGFPR01 (kindly supplied by Prof. Joan L. Slonczewski). Fecal supernatant with or without 20 mM L-lysine or L-ornithine was prepared to a final concentration of 50 mM acetate and 0.2% arabinose to induce GFP expression, and 50 μg/mL carbenicillin to maintain the plasmid. Media for a standard curve was prepared as described (38). Briefly, fecal supernatant was supplemented with 40 mM NaBenzoate and 40 mM methylamine, and aliquots were titrated to pH 5 to pH 8.5. Overnight cultures of *S.* Typhimurium transformed with pGFPR01 were pelleted, washed with MES-buffered saline, and diluted 1:10 in equilibrated 96-well plates. Optical density at 600 nm (OD_600_) was read concurrently with fluorescent emission at 510 nm following excitation at 405 nm and 488 nm every 30 min over a 20-h period in a microplate reader. Cytosolic pH measurements were calculated by comparing 510 nm emission ratios following excitation at 405 nm and 488 nm to those of the generated standard curve.

### Animal Experiments.

Upon arrival, 6- to 8-wk-old female CBA/J mice were maintained on 5058-PicoLab Mouse Diet 20 (LabDiet), an irradiated diet containing 20% protein and 9% fat. *S.* Typhimurium infections were performed by delivering 1 × 10^9^ CFU/mouse in 0.1 mL of LB broth intragastrically. Mock infections were performed by delivering 0.1 mL of sterile LB by broth intragastrically. For experiments using the C17 *Clostridium* consortium, each C17 strain was grown individually for 72 h anaerobically in Eggerth-Gagnon broth. Equal volumes of each culture were combined and used to gavage mice orally with 0.2 mL of the resulting mixture. Freshly voided fecal pellets were collected and bacterial burden was determined by homogenizing feces or cecal content in PBS and plating in 10-fold dilutions on LB agar (BD Biosciences) containing the appropriate antibiotics for selection. Where indicated, drinking water was prepared with 3% (w/v) L-lysine monohydrochloride, filter sterilized, and given to mice 24 h prior to infection. Fresh drinking water with lysine was prepared daily throughout the course of the experiment.

### RNA Isolation and qRT-PCR.

Murine enterocytes were isolated from freshly collected ileal tissue, defined as the last 10 cm of the small intestine immediately proximal to the cecum. Ileal tissue was cut longitudinally to expose the epithelial surface and then cut laterally into several pieces before being placed into ice-cold phosphate-buffered saline containing 0.5 M ethylenediaminetetraacetic acid (EDTA) and 1.5 mM dithiothreitol (DTT) for 20 min. Ileal tissue was then moved to phosphate-buffered saline containing 0.5 M EDTA and incubated at 37 °C for 10 min. Tubes containing ileal tissue were vigorously shaken by hand for approximately 30 s to release epithelial cells from the underlying tissue. Intact tissue was removed, and the resulting suspension of epithelial cells was centrifuged at 800×*g* for 5 min at 4 °C. The supernatant was aspirated, and the cell pellet was moved to a 2 mL screw cap microtube containing 1 mL of TRI Reagent (Molecular Research Center). Murine enterocytes were homogenized and lysed in a Mini-Beadbeater-16 (Biospec Products). Total RNA was isolated from the aqueous phase and subjected to on-column PureLink DNase (Invitrogen) digestion in EconoSpin (Epoch Life Science) columns before elution in UltraPure distilled water (Invitrogen). One microgram of isolated RNA was reverse transcribed with MultiScribe reverse transcriptase (Applied Biosystems) and random hexamers (Invitrogen) and RNase Inhibitor (Applied Biosystems) to form cDNA. Quantitative real-time PCR (qPCR) was performed using SYBR Green PCR master mix and the primers listed in *SI Appendix*, Table S3 on a ViiA 7 RT-PCR system (Applied Biosystems). The following parameters were used for reaction cycling: 50 °C for 2 min, 95 °C for 10 min, 40 cycles of 95 °C for 15 s, and 60 °C for 1 min. QuantiStudio Real-Time PCR software v1.3 (Applied Biosystems) and the 2^−ΔΔC^*^t^* method were used to calculate fold changes between experimental groups.

### Metabolomics Sample Collection and GC-TOF Mass Spectrometry.

Murine cecal contents were collected from mock-infected mice or mice on day 3 after *S.* Typhimurium infection. Samples were homogenized in sterile 0.9% NaCl at a final concentration of 250 mg/mL then centrifuged at 10,000×*g* for 5 min to pellet insoluble material and bacteria. The resulting supernatant was passed through a 10 kDa centrifugal filter (Millipore) to remove any remaining bacteria and then frozen at −80 °C. Frozen samples were transported on dry ice to the West Coast Metabolomics Center where they were processed for untargeted primary metabolite profiling by GC-TOF mass spectrometry. Data were obtained using the following parameters: column: Restek corporation Rtx-5Sil MS (30 m length × 0.25 mm internal diameter with 0.25 μm f ilm made of 95% dimethyl/5%diphenylpolysiloxane); mobile phase: helium; column temperature: 50 to 330 °C; flow rate: 1 mL/min; injection volume: 0.5 μL; injection: 25 splitless time into a multibaffled glass liner; injection temperature: 50 °C ramped to 250 °C by 12°C s^−1^; and oven temperature program: 50 °C for 1 min, then ramped at 20 °C min^−1^ to 330 °C, held constant for 5 min. The analytical GC column is protected by a 10-m long empty guard column which is cut by 20-cm intervals whenever the reference mixture QC samples indicate problems caused by column contaminations. This sequence of column cuts has been validated to have no detrimental effects with respect to peak shapes, absolute or relative metabolite retention times, or reproducibility of quantifications. Automatic liner exchanges are performed after each set of 10 injections which reduces sample carryover for highly lipophilic compounds such as free fatty acids. Mass spectrometry parameters are used as follows: a Leco Pegasus IV mass spectrometer is used with unit mass resolution at 17 spectra s^−1^ from 80 to 500 Da at −70 eV ionization energy and 1,800 V detector voltage with a 230 °C transfer line and a 250 °C ion source.

### Metabolomics Data Processing.

Raw data files are preprocessed directly after data acquisition and stored as ChromaTOF-specific *.peg files, as generic *.txt result files and additionally as generic ANDI MS *.cdf files. ChromaTOF vs. 2.32 is used for data preprocessing without smoothing, 3 s peak width, baseline subtraction just above the noise level, and automatic mass spectral deconvolution and peak detection at signal/noise levels of 5:1 throughout the chromatogram. Apex masses are reported for use in the BinBase algorithm. Result *.txt files are exported to a data server with absolute spectra intensities and further processed by a filtering algorithm implemented in the metabolomics BinBase database. The BinBase algorithm (rtx5) used the settings: validity of chromatogram (<10 peaks with intensity >10^7^ counts s^−1^), unbiased retention index marker detection (MS similarity > 800, validity of intensity range for high m/z marker ions), and retention index calculation by 5th order polynomial regression. Spectra are cut to 5% base peak abundance and matched to database entries from most to least abundant spectra using the following matching filters: retention index window ± 2,000 units (equivalent to about ± 2 s retention time), validation of unique ions and apex masses (unique ion must be included in apexing masses and present at > 3% of base peak abundance), mass spectrum similarity must fit criteria dependent on peak purity and signal/noise ratios, and a final isomer filter. Failed spectra are automatically entered as new database entries if s/n > 25, purity < 1.0, and presence in the biological study design class was > 80%. All thresholds reflect settings for ChromaTOF v. 2.32. Quantification is reported as peak height using the unique ion as default unless a different quantification ion is manually set in BinBase administration software BinView. A quantification report table is produced for all database entries that are positively detected in more than 10% of the samples of a study design class (as defined in the miniX database) for unidentified metabolites. A subsequent postprocessing module is employed to automatically replace missing values from the *.cdf files. Replaced values are labeled as “low confidence” by color coding, and for each metabolite, the number of high-confidence peak detections is recorded as well as the ratio of the average height of replaced values to high-confidence peak detections. These ratios and numbers are used for manual curation of automatic report datasets to datasets released for submission. These data were then normalized to the mTIC value (sum of the peak heights of the known metabolites). Normalized metabolomics spectra data are available in *SI Appendix*.

### Metabolomics Data Analysis.

Following processing, metabolomics data were analyzed using the shiny application, omuShiny (46). Principal component analysis was performed on metabolite abundances that were performed by the omuShiny glog function (ln(x + 1)). Ellipses denote a 95% CI on a multivariate t distribution. Univariate statistics were performed using ANOVA on ln(x + 1) transformed metabolite abundances followed by Tukey’s post hoc test. *P* values were adjusted using the Benjamini–Hochberg method to correct for the FDR (*SI Appendix*). Volcano plots were derived from data generated by the univariate statical analysis in omuShiny.

### Quantification of Lysine and ^13^C-Labeled Amino Acids.

#### Sample collection and preparation.

For quantification of fecal lysine, 2 to 3 murine fecal pellets were collected in 200 µL of PBS and kept on ice before being homogenized using a Vortex Genie 2 (Scientific Industries) equipped with a horizontal microtube holder (Scientific Industries). Homogenization was carried out at the maximum vortex intensity for 5 min or until complete homogenization of cecal contents was achieved. Samples were then centrifuged at 6,000×*g* for 10 min to pellet particulate matter. For each sample, 100 µL of the supernatant was combined with a solution of relevant internal standards. Samples were dried without heat in a vacuum dryer and then stored at −80 °C until use. For ^13^C-alanine/methionine/lysine quantification in serum, blood was collected from killed mice by cardiac puncture using a 26G, 13 mm needle and then incubated at room temperature for 1 h prior to centrifugation for 15 min at 3,000 rpm to pellet red blood cells. Then, 50 µL of serum was collected and combined with a solution of relevant internal standards. Proteins were precipitated by adding 250 µL of methanol to each sample and vortexing for 60 s. Samples were then centrifuged for 10 min at 17,000×*g* and all the supernatant was recovered, then dried without heat in a vacuum dryer and stored at −80 °C until use.

Dried extracts were solubilized by sonication in 50 µL anhydrous pyridine (Sigma Aldrich) and then incubated for 20 min at 80 °C. An equal amount of N-tert-butyldimethylsilyl-N-methyltrifluoroacetamide with 1% tert-butyldimethylchlorosilate (Sigma-Aldrich) was added, and the samples were incubated for 1 h at 80 °C. Samples were centrifuged at 17,000×*g* for 1 min to remove leftover particles. Around 70 µL of the supernatant was transferred to an autosampler vial and analyzed by gas chromatography–mass spectrometry (Agilent 8890 Gas Chromatograph and Agilent 7000D Mass spectrometer).

#### GC–MS/MS Analysis.

For all experiments, 1 µL of the sample was injected with a 1:50 split ratio at an injection temperature of 250 °C on an HP 5 ms Ultra Inert (2 × 15-m-length, 0.25-mm diameter, 0.25 µm film thickness) fused silica capillary column. Helium was used as the carrier gas with a constant flow of 1.2 mL/min. The interface was heated to 300 °C and the ion source was used in electron ionization (EI) mode (70 V, 150 µA, 230 °C). Efficient recovery of target metabolites was determined using deuterated compounds as internal standards. Quantification was based on external standards composed of a series of dilutions of pure compounds, derivatized as described above at the same time as the samples. For L-Lysine analysis, the GC oven temperature started at 50 °C, rising to 290 °C at 15 °C/min, then to 310 °C at 40 °C/min with a final hold for 2 min. The dwell time for multiple reaction monitoring (MRM) events was 25 ms. Analytes were quantified using MRM, with the monitored transitions and experimentally determined retention times detailed *SI Appendix*, Table S4. For ^13^C amino acids analysis, the GC oven temperature started at 50 °C for 1 min, rising to 310 °C at 10 °C/min with a final hold for 3 min. Analytes were quantified using MRM, with the monitored transitions, experimentally determined retention times, and dwell times detailed in *SI Appendix*, Table S4.

## Supplementary Material

Appendix 01 (PDF)

Dataset S01 (XLSX)

## Data Availability

All study data are included in the article and/or supporting information. Previously published data were used for this work (42).
