# Supplementary material for: Salmonella virulence factors induce amino acid malabsorption in the ileum to promote ecosystem invasion of the large intestine
Source: Proc Natl Acad Sci U S A. 2024 Nov 15;121(47):e2417232121. doi: 10.1073/pnas.2417232121 (PMC11588050; doi:10.1073/pnas.2417232121)
Supplement: Supplementary file 1 — Appendix 01 (PDF) [file pnas.2417232121.sapp.pdf]

SUPPLEMENTAL FIGURES AND TABLES

Supplemental file 1: Excel-file containing metabolite spectra data and statistical anlysis.

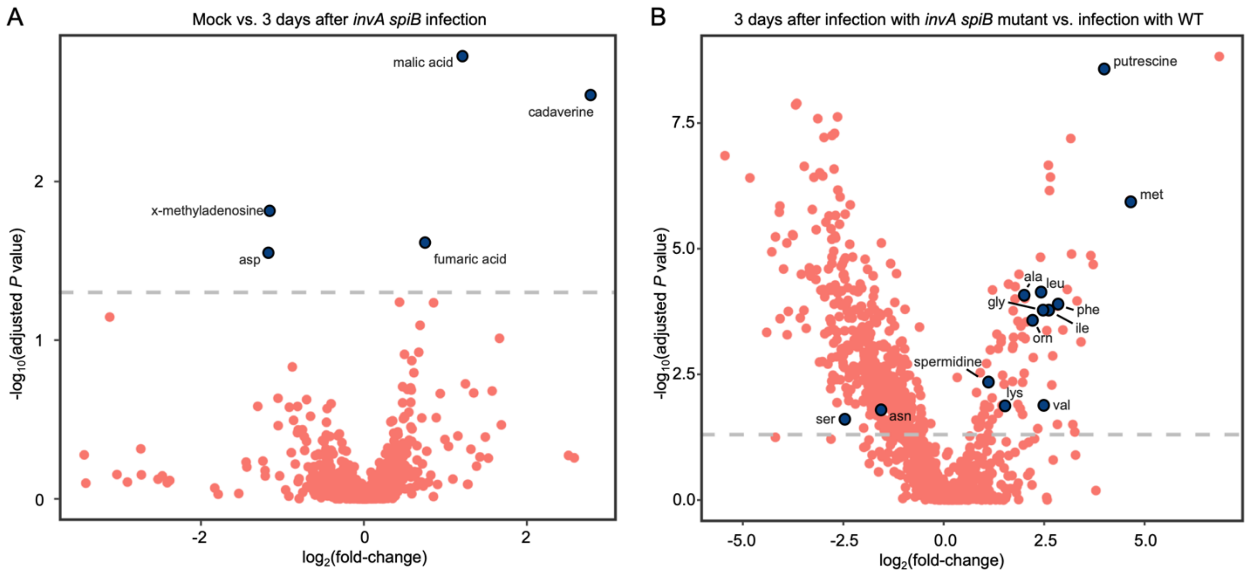

Figure S1: ***S.* Typhimurium virulence factors change the cecal metabolome.**

Germ-free Swiss Webster mice were engrafted with a defined microbial consortium consisting of 17 human *Clostridia* isolates(1). One week later, mice were mock infected (mock) (*N* = 5), infected with the *S.* Typhimurium wild type (WT) (*N* = 8), or with an avirulent *S.* Typhimurium *invA spiB* mutant (*N* = 6). Cecal contents were collected three days after infection for untargeted metabolomics analysis. Volcano blots showing metabolite abundance in the ceca of mock-infected mice vs. *invAspiB*-infected mice (A) or *invAspiB*-infected mice vs WT-infected mice (B). The Y-axis shows the decadic logarithm of the false discovery rate (FDR)-corrected *P* value. The dashed line is set at an FDR corrected *P* value of 0.05. (A) Metabolites with a negative fold-change value decreased in mice infected with *a invA spiB* mutant, while metabolites with a positive fold-

change value increased. (B) Metabolites with a negative fold-change value decreased in mice infected with the *S.* Typhimurium wild type, while metabolites with a positive fold-change value increased.

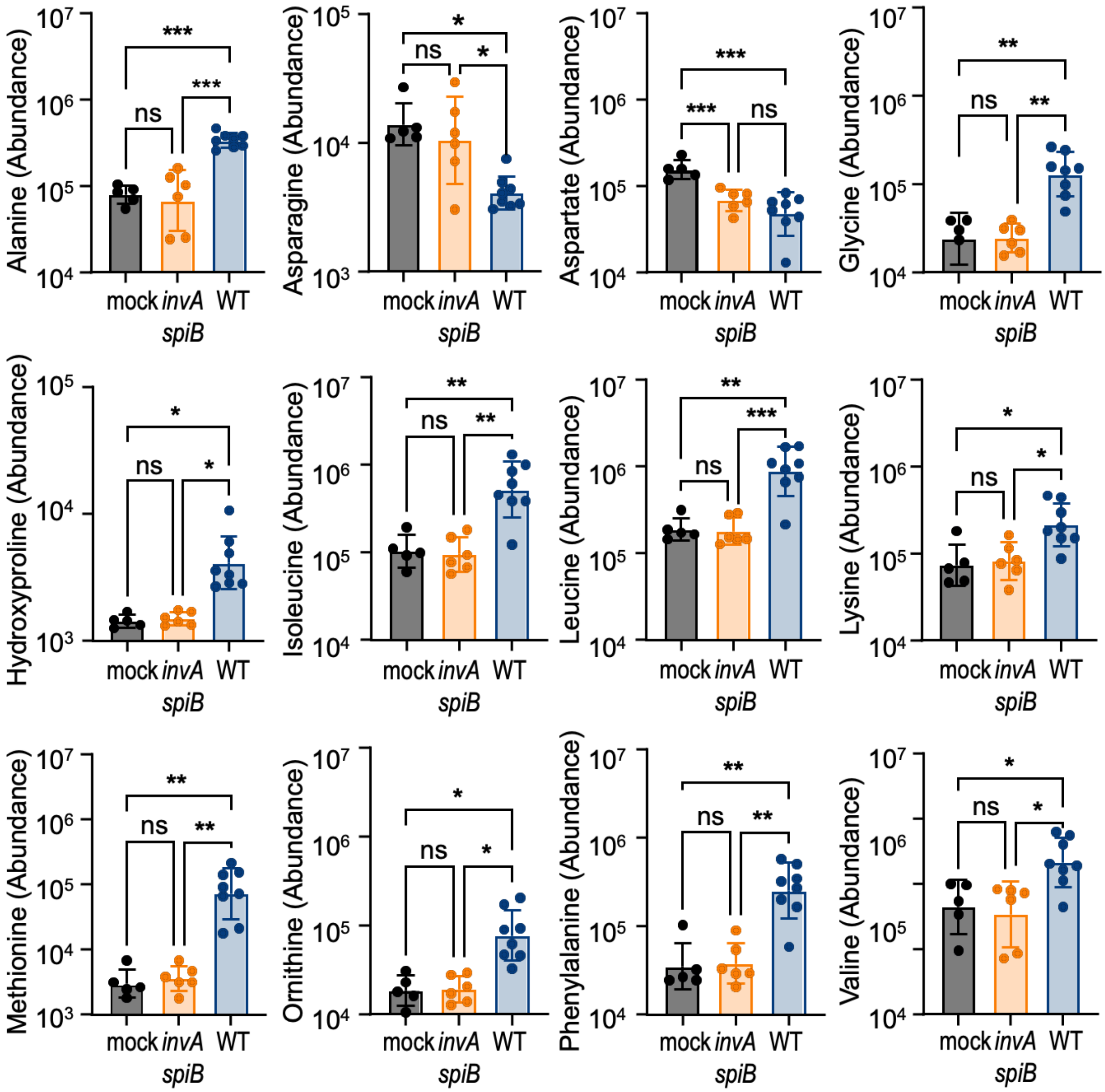

Figure S2: *S.* Typhimurium virulence factors increase the concentrations of amino acids in the cecum.

Germ-free Swiss Webster mice were engrafted with a defined microbial consortium consisting of 17 human *Clostridia* isolates(1). One week later, mice were mock infected (mock) (*N* = 5), infected with the *S.* Typhimurium wild type (WT) (*N* = 8), or with an avirulent *S.* Typhimurium *invA spiB* mutant (*N* = 6). Cecal contents were collected three

days after infection for untargeted metabolomics analysis. The graph shows the mean abundance of the indicated amino acids in the cecal contents of mock-infected mice or mice infected with the indicated *S.* Typhimurium strains. Each symbol represents data from one animal. Bars represent geometric mean ± standard error. *, $P < 0.05$; **, $P < 0.01$; ***, $P < 0.001$; ns, not significant; (One-way ANOVA with Tukey's multiple comparison test).

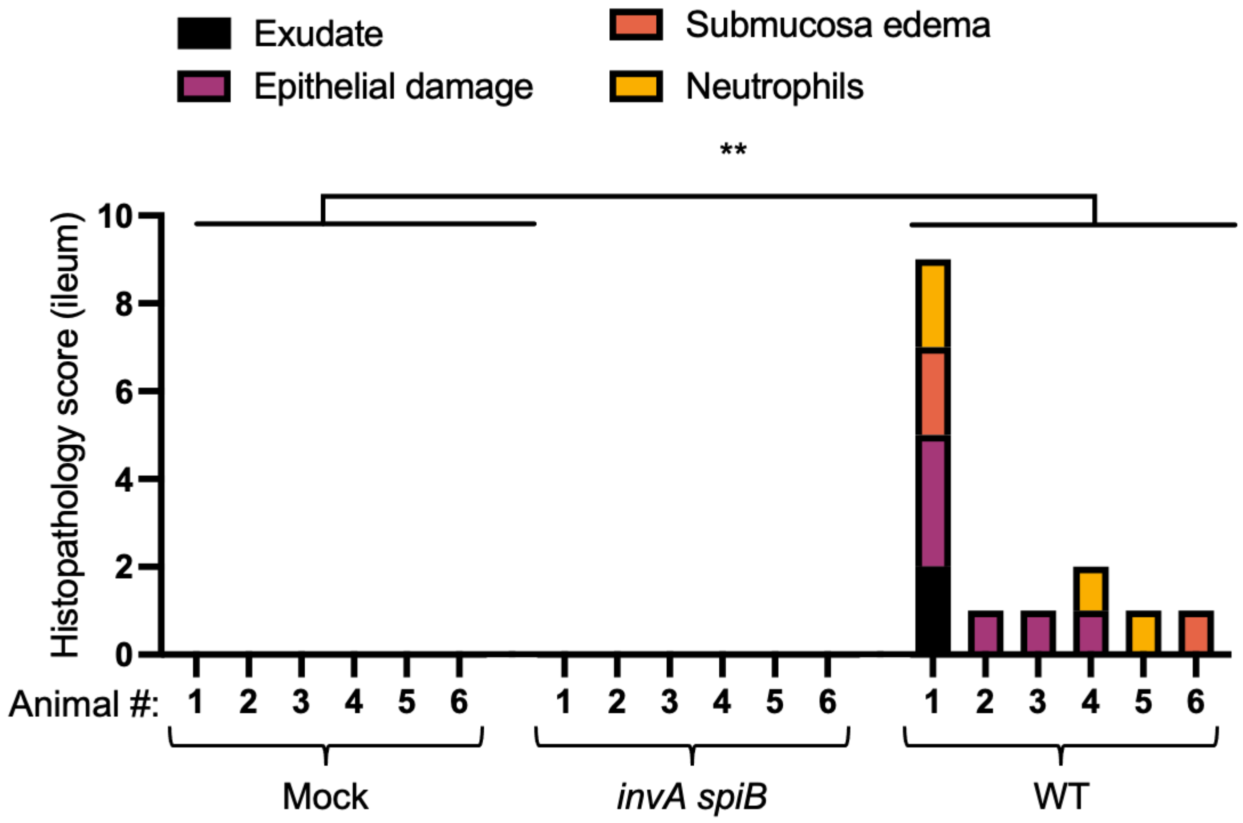

Figure S3: ***S.* Typhimurium causes inflammatory changes in the ileum.**

Groups of CBA/J mice (*N* = 6) were mock infected (mock), or infected with 10^9 CFU of the *S.* Typhimurium wild type (WT) or an isogenic *S.* Typhimurium *invAspiB* mutant (*invA spiB*). Four days after infection, tissue was collected and blinded sections of the ileum were scored by a veterinary pathologist. **, P<0.001 (Kruskal-Wallis with Dunn's multiple comparisons post-test).

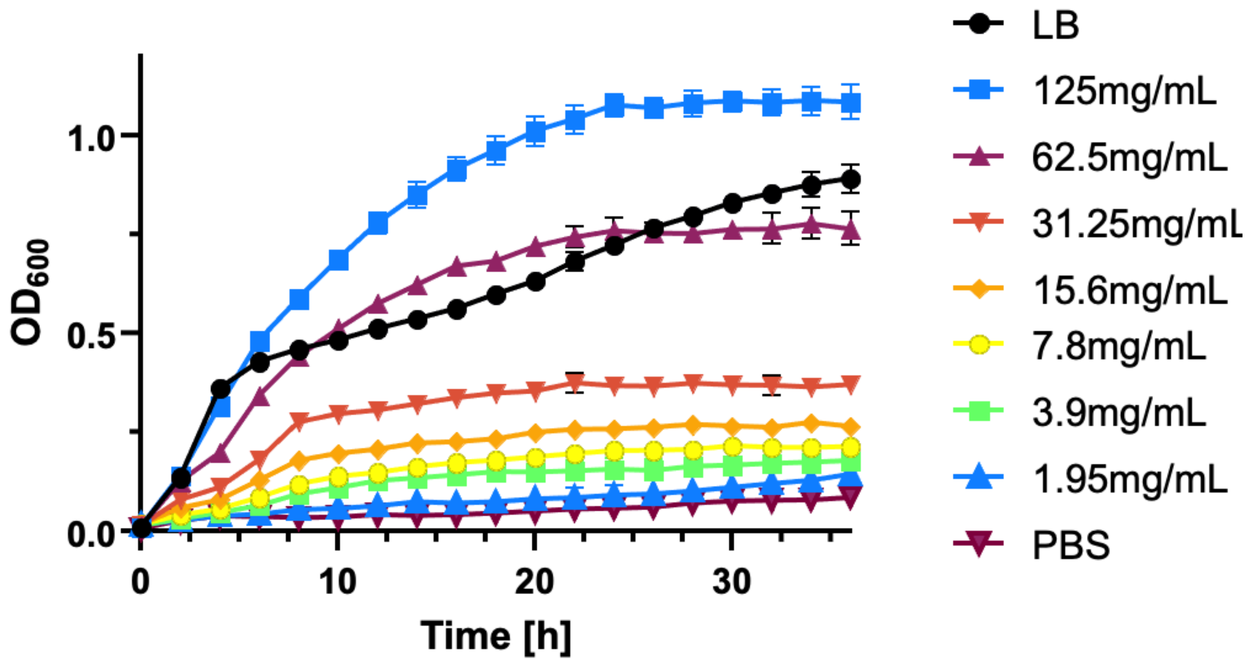

Figure S4. Fecal homogenate media was prepared by homogenizing fecal pellets from untreated mice in PBS (pH 7.8) , and equilibrating the sterilized supernatant to 0.5-1% O_2. *S.* Typhimurium growth in LB or fecal homogenate media prepared at the indicated concentrations was monitored by quantifying absorbance (OD_{600}) over a 36-hour period.

Table S1: Bacterial strains and plasmids used in this study.

**Bacterial Strains**		
**Species and strain**	**Genotype**	**Reference**
E. coli DH5α l λ*pir*	F$^-$ endA1 *hsdR17* (r^-m^+) *supE44 thi-1 recA1 gyrA relA1* Δ*(lacZYA-argF)U189* Φ80l*acZ*Δ*M15* λ*pir*	(2)
E. coli S17-1 λ*pir*	zxx::RP4 2-(Tet^R::Mu) (Kan^R::Tn7) λ*pir recA1 thi pro hsdR* (r^-m^+)	(3)
S. Typhimurium IR715	ATCC14028 Nal^R (Nal^R)	(4)
S. Typhimurium FF176	ATCC14028 Nal^R *phoN*::Tn*10dCam* (Nal^R, Cm^R)	(5)
S. Typhimurium AJB715	ATCC14028 Nal^R *phoN*::KSAC (Nal^R, Kan^R)	(6)
S. Typhimurium FF183	ATCC14028 Nal^R Δ*invA* Δ*spiB phoN*::Tn*10dCam* (Nal^R, Cm^R)	(7)
S. Typhimurium FF459	ATCC14028 Nal^R Δ*invA* Δ*spiB phoN*::KSAC (Nal^R, Kan^R)	(8)
S. Typhimurium LR82	ATCC14028 Nal^R Δ*cadBA phoN*::KSAC (Nal^R, Kan^R)	This Study

S. Typhimurium LR81	ATCC14028 Nal^R Δ*speFpotE phoN*::KSAC (Nal^R, Kan^R)	This Study
**Plasmids**		
**Name**	**Genotype**	**Reference**
pRE112	*sacB1 cat ori*R6K *mobRP4*	(9)
pRE112-*cadBA*	pRE112 with upstream and downstream regions of *S.* Typhimurium *cadBA* forming a deletion mutant allele	This Study
pRE112-*speFpotE*	pRE112 containing upstream and downstream regions of *S.* Typhimurium *speFpotE* forming a deletion mutant allele	This Study
pGFP01	Arabinose-inducible pHluorin expression construct	(10)

Table S2: Primers used for targeted mutagenesis in this study.

Primers used for Targeted Mutagenesis		
**Target**	**Sequence**	**Reference**
cadBA	5'-AACGCCATTCATGGCCATATCAATGCGCTGATGCCTATCTGAC-3'<br>5'-GGGCGCCGATTTAACTCATGTTCATTTCTCCTG-3'<br>5'-ATGAACATGAGTTAAATCGGCGCCCACTATC-3'<br>5'-TCCCGGGAATTCATGCAGTTCACTTCCGCAGATAATAATGGCCAG-3'	This Study
speFpotE	5'-AACGCCATTCATGGCCATATCAATGACAGATTGCTGGCGCTTC-3'<br>5'-CAGGCCGTATAAATCCGGGCAATGACGGCT-3'<br>5'-TCATTGCCCGGATTTATACGGCCTGGTCTCAC-3'<br>5'-TCCCGGGAATTCATGCAGTTCACTTACGCATAATGACGTCCAG-3'	This Study
pRE112 (pRE_linear_F)	5'-AAGTGAACTGCATGAATTC-3'	*(11)*
pRE112 (pRE_linear_R)	5'- CATTGATATGGCCATGAATG -3'	*(11)*
pRE112 (pRE_chk_F)	5'-CGTAAAATCGTGTTGAGGCC-3'	*(11)*

pRE112<br><br>(pRE_chk_R)	5'-AGCTTCTTCTAGAGGTACCGC-3'	*(11)*

Table S3: Primers used for quantitative real-time PCR in this study.

**Primers used for qRT-PCR**			
**Target gene**	**Sequence**	**Organism**	**Reference**
β2m	5'-GGTCTTTCTGGTGCTTGTCTCA-3'<br>5'-GTTCGGCTTCCCATTCTCC-3'	Mus musculus	(12)
Lcn2	5'-ACATTTGTTCCAAGCTCCAGGGC-3'<br>5'-CATGGCGAACTGGTTGTAGTCCG-3'	Mus musculus	(13)
slc6a19	5'-CATCCACCCTGCTCTGAAGG-3'<br>5'-CACACTCTTCCACATAGCCTGTC-3'	Mus musculus	This Study
slc3a1	5'-CCCAACAACTGGCTGAGTGT-3'<br>5'-CAACACCCTTCGAGAGCCAG-3'	Mus musculus	This Study
slc1a1	5'-TTTCTCCACCACCGTCATTGC-3'<br>5'-CCCGCTTGGTTTTGTACTGCTG-3'	Mus musculus	This Study
slc7a9	5'-ACTCTGGGCCTACGATGGTT-3'<br>5'-GGTCTCCGAAGGTCACAGC-3'	Mus musculus	This Study

Table S4: GC-MS/MS retention times, quantifiers and dwell times.

Analyte	Retention time (minutes)	Quantifier (m/z)	Qualifier (m/z)	Dwell time (ms)
L-Lysine	14.70	300->147	300->168	-
L-Lysine-d4	14.69	303->147	303->171	-
L-Alanine-^{13}C3	13.21	160->73	234->147	40
L-Methionine-^{13}C5	17.89	296->147	222->173	25
L-Lysine-^{13}C6	21.59	306-147	306->174	25
L-Leucine-d7	15.03	281->147	309->281	50
L-Lysine-d4	21.58	303->147	303->171	25

REFERENCES

1. **Atarashi, K., T. Tanoue, T. Shima, A. Imaoka, T. Kuwahara, Y. Momose, G. Cheng, S. Yamasaki, T. Saito, Y. Ohba, T. Taniguchi, K. Takeda, S. Hori, Ivanov, II, Y. Umesaki, K. Itoh, and K. Honda.** 2011. Induction of colonic regulatory T cells by indigenous Clostridium species. Science **331:**337-41.
2. **Pal, D., T. Venkova-Canova, P. Srivastava, and D. K. Chattoraj.** 2005. Multipartite regulation of rctB, the replication initiator gene of Vibrio cholerae chromosome II. J Bacteriol **187:**7167-75.
3. **Simon, R., U. Priefer, and A. Puhler.** 1983. A Broad Host Range Mobilization System for Invivo Genetic-Engineering - Transposon Mutagenesis in Gram-Negative Bacteria. Bio-Technology **1:**784-791.
4. **Stojiljkovic, I., A. J. Baumler, and F. Heffron.** 1995. Ethanolamine utilization in Salmonella typhimurium: nucleotide sequence, protein expression, and mutational analysis of the cchA cchB eutE eutJ eutG eutH gene cluster. J Bacteriol **177:**1357-66.
5. **Faber, F., L. Tran, M. X. Byndloss, C. A. Lopez, E. M. Velazquez, T. Kerrinnes, S. P. Nuccio, T. Wangdi, O. Fiehn, R. M. Tsolis, and A. J. Baumler.** 2016. Host-mediated sugar oxidation promotes post-antibiotic pathogen expansion. Nature **534:**697-9.
6. **Kingsley, R. A., A. D. Humphries, E. H. Weening, M. R. De Zoete, S. Winter, A. Papaconstantinopoulou, G. Dougan, and A. J. Baumler.** 2003. Molecular and phenotypic analysis of the CS54 island of Salmonella enterica serotype typhimurium: identification of intestinal colonization and persistence determinants. Infect Immun **71:**629-40.
7. **Faber, F., P. Thiennimitr, L. Spiga, M. X. Byndloss, Y. Litvak, S. Lawhon, H. L. Andrews-Polymenis, S. E. Winter, and A. J. Baumler.** 2017. Respiration of Microbiota-Derived 1,2-propanediol Drives Salmonella Expansion during Colitis. PLoS Pathog **13:**e1006129.
8. **Walker, G. T., G. Yang, J. Y. Tsai, J. L. Rodriguez, B. C. English, F. Faber, L. Souvannaseng, B. P. Butler, and R. M. Tsolis.** 2021. Malaria parasite infection compromises colonization resistance to an enteric pathogen by reducing gastric acidity. Sci Adv **7**.
9. **Edwards, R. A., L. H. Keller, and D. M. Schifferli.** 1998. Improved allelic exchange vectors and their use to analyze 987P fimbria gene expression. Gene **207:**149-57.
10. **Martinez, K. A., 2nd, R. D. Kitko, J. P. Mershon, H. E. Adcox, K. A. Malek, M. B. Berkmen, and J. L. Slonczewski.** 2012. Cytoplasmic pH response to acid stress in individual cells of Escherichia coli and Bacillus subtilis observed by fluorescence ratio imaging microscopy. Appl Environ Microbiol **78:**3706-14.
11. **Rogers, A. W. L., L. C. Radlinski, H. Nguyen, C. R. Tiffany, T. Parente Carvalho, H. L. P. Masson, M. L. Goodson, L. Bechtold, K. Yamazaki, M. J. Liou, B. M. Miller, S. P. Mahan, B. M. Young, A. M. Demars, S. R. Gretler, A. B. Larabi, J.-Y. Lee, D. J. Bays, R. M. Tsolis, and A. J. Bäumler.** 2024. Salmonella re-

engineers the intestinal environment to break colonization resistance in the presence of a compositionally intact microbiota. Cell Host & Microbe **32:**In press.

12. **Matouskova, P., H. Bartikova, I. Bousova, V. Hanusova, B. Szotakova, and L. Skalova.** 2014. Reference genes for real-time PCR quantification of messenger RNAs and microRNAs in mouse model of obesity. PLoS One **9:**e86033.
13. **Godinez, I., T. Haneda, M. Raffatellu, M. D. George, T. A. Paixao, H. G. Rolan, R. L. Santos, S. Dandekar, R. M. Tsolis, and A. J. Baumler.** 2008. T cells help to amplify inflammatory responses induced by Salmonella enterica serotype Typhimurium in the intestinal mucosa. Infect Immun **76:**2008-17.
